# American crocodiles (*Crocodylus acutus*: Reptilia: Crocodilidae) visiting the facilities of a freshwater aquaculture of the Northern Pacific region, Costa Rica, carry tetracycline-resistant *Escherichia coli*

**DOI:** 10.3389/fvets.2024.1374677

**Published:** 2024-04-05

**Authors:** Rafael Hernán Mateus-Vargas, Verónica Arias-Pérez, Iván Sandoval-Hernández, Jens Andre Hammerl, Elías Barquero-Calvo

**Affiliations:** ^1^Department of Animal Sciences, University of Göttingen, Göttingen, Germany; ^2^Escuela de Ciencias Biológicas, Universidad Nacional, Heredia, Costa Rica; ^3^Department Biological Safety, German Federal Institute for Risk Assessment, Berlin, Germany; ^4^Programa de Investigación en Enfermedades Tropicales, Escuela de Medicina Veterinaria, Universidad Nacional, Heredia, Costa Rica

**Keywords:** antimicrobial susceptibility, human activities, human-animal-environment interface, captivity, whole-genome sequencing, plasmid carriage

## Abstract

Apex predators are exposed to antimicrobial compounds and resistant microbes, which accumulate at different trophic levels of the related ecosystems. The study aimed to characterize the presence and the antimicrobial resistance patterns of fecal *Escherichia coli* isolated from cloacal swab samples obtained from wild-living American crocodiles (*Crocodylus acutus*) (*n* = 53). Sampling was conducted within the distinctive context of a freshwater-intensive aquaculture farm in Costa Rica, where incoming crocodiles are temporarily held in captivity before release. Phenotypic antimicrobial susceptibility profiles were determined in all isolates, while resistant isolates were subjected to whole-genome sequencing and bioinformatics analyses. In total, 24 samples contained tetracycline-resistant *E. coli* (45.3%). Isolates carried either *tet*(A), *tet*(B), or *tet*(C) genes. Furthermore, genes conferring resistance to ß-lactams, aminoglycosides, fosfomycin, sulfonamides, phenicol, quinolones, trimethoprim, and colistin were detected in single isolates, with seven of them carrying these genes on plasmids. Genome sequencing further revealed that sequence types, prevalence of antibiotic resistance carriage, and antibiotic resistance profiles differed between the individuals liberated within the next 24 h after their capture in the ponds and those liberated from enclosures after longer abodes. The overall presence of tetracycline-resistant *E. coli*, coupled with potential interactions with various anthropogenic factors before arriving at the facilities, hinders clear conclusions on the sources of antimicrobial resistance for the studied individuals. These aspects hold significant implications for both the aquaculture farm’s biosecurity and the planning of environmental monitoring programs using such specimens. Considering human-crocodile conflicts from the One Health perspective, the occurrence of antimicrobial resistance underscores the importance of systematical surveillance of antibiotic resistance development in American crocodiles.

## 1 Introduction

Globally, the development of antimicrobial resistance (AMR) in bacteria is a growing public health concern because the number of antimicrobial substances for effectively treating bacterial infections decreases rapidly in human and veterinary medicine. Since their introduction for medical application in the 1940s, antimicrobial compounds have contributed to spreading resistant microbes in different ecosystems and increasing the complexity of their resistomes (1). From the One Health perspective, the spread of AMR poses an important risk to humans, animals, and the environment (2).

Regarding the environment, apex predators are considered appropriate sentinels to evaluate the general health of natural ecosystems (3). For instance, the ecological role of such species has been used to analyze the occurrence of chemical and biological contaminants, including AMR, in wildlife. The latter due to the trophic transfer and bioaccumulative tendency of such agents along the trophic chain in natural as well as in human-influenced ecosystems (4–14). Regarding the presence of AMR in wild populations of predators of the American continent, studies using culture or direct genetic approaches confirmed the presence of indicator bacteria with AMR or antibiotic resistance genes (ARGs) in faecal samples of wild felids (10, 15–17), and foxes (16, 18). However, to our knowledge, no other published data is available regarding the epidemiological status of AMR in terrestrial, water-associated apex predators of the continent. The latter except the previous reports on opportunistic predators, such as raccoons (*Procyon lotor*) by Bondo et al. (19) or Vogt et al. (20) in Canada as well as by Baldi et al. (21) in Costa Rica. In the tropics and subtropics, crocodilians are prominent representatives of the highest trophic levels of freshwater and coastal ecosystems (22). They may have a unique role in surveys and monitoring efforts on the occurrence of AMR in wildlife (6).

In conjunction with the geographic expansion of human activities, human-wildlife conflicts are a growing problem worldwide (23). Crocodilians are one of the major groups involved in regions in which they occur (24). Different studies in Mesoamerica showed that anthropic activities, such as livestock, aquaculture productions, fisheries, and crops, generate high pressure on the natural freshwater habitats of crocodiles (25–27). The reduction and fragmentation of habitats by human influence, as well as the increase of “alternative” feed sources (e.g., domestic animals), have led the native crocodile populations to move to habitat sites of lower quality, such as crop irrigation channels, streams, intermittent ponds, flooded lagoons, fisheries, and aquaculture farms, or even to human settlements (26–28). Such regional displacements have resulted in an increasing frequency of interactions between humans and crocodiles, which has additionally increased the number of non-fatal and fatal attacks on residents of the local communities (28–32). Risks may be potentiated by the lack of knowledge of local communities and the deliberate direct contact, for instance, linked to the exploitation of wild populations (33). In this matter, it is important to consider that crocodiles may carry important zoonotic pathogens (9, 31), and bite injuries caused by them are especially challenging for their treatment in humans, as reported in the African and American continent (29, 30, 34).

In Costa Rica, the local populations of crocodiles, especially those of *Crocodylus* (*C.*) *acutus*, have grown substantially due to the protection status provided by the law N° 7,317 for the conservation of wildlife in Costa Rica (35). This species is a generalist with a broad preying and scavenging mixed diet, composed of arthropods, snails, crustaceans, turtles, and mammals, although its favorite prey is fish (36, 37). Therefore, the presence of bodies of water, a dense drainage network, and the existence of mangroves and estuaries in altitudes below 240 meters above sea level (masl) significantly contribute to the occurrence and persistence of crocodiles in Costa Rican’s Pacific and Caribbean regions (26, 27). Additionally, the expansion of forest coverage and the growth of the human population through settlement and agriculture seem to locally amplify the availability of habitat for crocodiles, thereby leading to an overall increase in their abundance (26, 27). Linked to the industrialization of aquaculture farming in the country, a marked increase in wild populations in regions with a high density of aquaculture production has been additionally reported (38). Due to the favorable climate conditions and the irrigation system Arenal-Tempisque, the most important aquaculture producers are located in the Northwest region of Costa Rica, where fish is mainly farmed in earthen ponds (39). The area of the earthen ponds is generally fenced but not isolated from the surroundings. The ponds with the concentrated fish stock represent a highly attractive feed source for crocodiles and other wildlife. Therefore, crocodiles regularly enter aquaculture farms and settle in ponds, often reaching considerable numbers (38). Such animals inhabit and cross ecosystems with different gradients of human influence during their life, including settlements and agricultural land, before entering the aquaculture premises. After, they are directly or indirectly in contact with the aquaculture environment for hours, weeks, or even months until they are released into protected natural reserves (38). Hence, epidemiological information regarding the existence and subsequent development of AMR within the microbiota of these individuals, reflected in indicator microorganisms such as *Escherichia coli*, holds significant importance in identifying potential hazards for both public and environmental health.

This study aims to evaluate the antimicrobial resistant determinants in *E. coli* isolates obtained from American crocodiles (*C. acutus*) captured in a commercial freshwater aquaculture farm in Costa Rica for release. Phenotypic antimicrobial susceptibility profiles were performed in all isolates. In addition, resistant isolates were subjected to whole-genome sequencing and bioinformatics analyses.

## 2 Materials and methods

### 2.1 Study area and sample collection

Fifty-three American crocodile (*C. acutus*) individuals, which entered the facilities of an intensive aquaculture farm in the North Pacific region of Costa Rica between November 2017 and December 2018, were sampled for microbiological examination. Sampling was conducted in accordance with the crocodile management plan approved by the National System of Conservation Areas of Costa Rica (SINAC). The American crocodile is classified as vulnerable in the IUCN Red List, listed on Appendix II of CITES, and identified as endangered under Costa Rica’s Wildlife Conservation Law (7317) (35). At the time of the study, the management plan had received approval from local authorities and was carried out in collaboration with the aquaculture company (M-P-SINAC-PNI-ACAT-041-2021). All relevant institutional and national guidelines for the ethical care, welfare, and use of animals were followed during the capture, sampling, and transportation of individuals as outlined in the management plan. The handling of animals, including sampling procedures, was explicitly detailed in the management plan, obviating the need for additional ethical approval for the study. For further information, capture and release activities are thoroughly described by Bolaños (38).

From the geographic perspective, at an elevation of 85 masl, North Pacific region is characterized by its tropical dry forests, temperature average of 27°C and an average annual rainfall of 2,000 mm (35). Besides, this area has the country’s most extensive network of irrigation channels for agriculture and aquaculture. These beneficial characteristics are used by the inland aquaculture producer, which manages up to 600 hectares of earthen ponds in the middle basin of the Tempisque River. Due to frequent break-in episodes, the company initiated a management plan in which the crocodiles were captured in the fish production ponds to be later released in a national park (38). Disregarding the sex, animals were removed from production ponds shortly after being spotted on the premises. Following specific requirements of the management plan, females were liberated within the next 24 h after removal from ponds, while males were first relocated to an enclosure of the aquaculture farm for their maintenance for weeks or months until their release, either with females or at the end of the year (38). Bolaños (38) described that individuals were immobilized without using medicaments for liberation and transported to protected natural reserves, such as the National Park Palo Verde (approximately 40 km). After animal restraining, animal data regarding sex, body size, and identification number (microchip identification) were protocolled. Subsequently, cloacal samples of each captured animal were obtained using a sterile swab, previously moistened with 0.85% saline solution. After sampling, the swabs were immediately introduced into Amies with charcoal transport media (Oxoid) and transported within 12 h at 9°C ± 1°C to the Bacteriology Laboratory of the Veterinary School of the Universidad Nacional.

### 2.2 Detection of resistant *Escherichia coli*

Once in the laboratory, swab samples were incubated for 20–24 h at 35°C ± 1°C in 5 mL of enriching liquid medium (EC broth; Oxoid). After enrichment, samples were cultured on MacConkey agar plates with or without antibiotics. Due to the explorative character of the study, adjustments regarding the selective antimicrobial substances were implemented during the study period. Namely, the first 16/53 samples were solely streaked on non-supplemented MacConkey agar or supplemented with cefotaxime (2 mg/L) (Sigma) to detect resistance to 3rd generation cephalosporins. Due to the failure to find cefotaxime-resistant bacteria in all these first samples, we also included the use of plates supplemented with tetracycline (16 mg/L) (Sigma) for the rest of the obtained samples (37/53). Antimicrobial concentrations for selective enrichment were chosen in agreement with the interpretive categories and MIC breakpoints of the CLSI M100 manual for *Enterobacteriaceae* to detect resistant *E. coli* (Clinical and Laboratory Standards Institute, 30th Edition; (40)). Following 20–24 h incubation at 35°C ± 1°C, agar plates were inspected for colonies showing typical morphology for *E. coli* (red-pink colonies). For the growth of suspicious *E. coli* colonies on agar plates supplemented with antimicrobials, a colony was randomly selected for each substance. If there was no growth on supplemented media, a colony was selected from the plates without antibiotics. Selected colonies were cultured on trypticase soy agar (BD) and incubated for 24 h at 35°C ± 1°C. Pure cultures were biochemically confirmed as *E. coli* with the Vitek 2 Compact system using the GN identification card following the manufacturer’s recommendations (bioMérieux). Confirmed isolates were suspended in duplicate 1 mL aliquots in sterile skim milk (10%) with sterile glycerol (20%) and frozen at −80°C for further analysis. As a reference for the media quality control, *E. coli* ATCC 25922, *Klebsiella pneumoniae* ATCC 33495 and *Pseudomonas aeruginosa* ATCC 27853 were used to exclude growth on antimicrobial supplemented agar plates and confirm typical growth on non-supplemented media.

### 2.3 Antimicrobial susceptibility testing

The susceptibility of the isolates to different antimicrobial substances was determined with the Vitek 2 system using the AST-N279 card (bioMérieux) following the manufacturer’s recommendations. This VITEK card includes the following antimicrobial substances: amikacin, ampicillin, ampicillin/sulbactam, cephalothin, cefazolin, cefepime, cefotaxime, ceftazidime, ceftriaxone, ciprofloxacin, colistin, fosfomycin, gentamicin, imipenem, ertapenem, meropenem, nalidixic acid, nitrofurantoin, piperacillin/tazobactam, and trimethoprim/sulfamethoxazole.

Minimal Inhibitory Concentration (MIC) for tetracycline-resistant isolates was measured using the ETEST^®^ method (bioMérieux) following the manufacturer’s recommendation. Briefly, a fresh suspension of the tetracycline-resistant *E. coli* isolate was prepared in saline solution at 0.5 McFarland. Subsequently, each suspension was streaked on Mueller Hinton agar (Oxoid), and finally, an ETEST strip was placed on the middle of the plate and incubated for 18–24 h at 35°C ± 1°C. MIC was read following the manufacturer’s specifications and interpreted according to the CLSI M100 manual (30th Edition, (40)).

### 2.4 Whole-genome sequencing and data analysis

WGS was conducted with genomic DNA isolated using the Wizard^®^ Genomic DNA Purification kit (Promega) according to the manufacturer’s instructions. DNA sequencing libraries were prepared using the Nextera DNA Flex library prep kit, and paired-end sequencing (2 × 151 bp) was conducted on a NextSeq 500 (Illumina, Inc., San Diego, CA, United States) (41). Raw reads were trimmed and subjected to the Aquamis pipeline for genome assembly (https://gitlab.com/bfr_bioinformatics/AQUAMIS/, access date: February 2023) (42). Bacterial genome annotation was performed using the automated Prokaryotic Genome Annotation Pipeline (PGAP, National Center for Biotechnology Information) (43). SNP-based phylogenetic analysis of genome sequences was performed using CSIPhylogeny 1.4 (default settings) with isolate 23-MO00003 as a reference (44). The phylogenetic analysis uses Burrows-Wheeles Aligner (BWA), SAMtools, and BEDtools for nucleotide sequence alignments followed by SNP calling, MUMmer to assess genome similarities, and FastTree 2 for maximum likelihood tree estimation (45). *In silico* (sub) typing was conducted with the bioinformatics pipeline Bakcharak (41).

Genome sequences were deposited in the GenBank database (https://www.ncbi.nlm.nih.gov/genbank/accessed on 13 October 2023) under bioproject PRJNA1027614, and accession numbers for all isolates are listed in Supplementary Table S1.

### 2.5 Statistical analyses and clustering

Initially, descriptive statistics were performed regarding the detection rate of *E. coli* for all animals separated by sex, age class, and management type (liberation directly from ponds or after temporary captivity in the enclosure). Following the classification scheme published by Sandoval-Hernández et al. (27), sampled animals were categorized as juveniles or adults based on their body size (< 2.0 m and ≥ 2.0 m, respectively). Fisher’s exact test was calculated to determine differences in detection frequencies of tetracycline-resistant *E. coli* in cloacal swabs between animals of different sexes, age classes, and animals sampled of different management types before release. The results of the statistical tests were considered significant for *p* < 0.05. Statistical calculations were performed using SAS software version 9.3 for Windows (SAS Institute Inc., Cary, NC, United States).

## 3 Results

### 3.1 Number of isolates and antimicrobial susceptibility profiles

Of the sampled animals, 21 were females (body size range: 168–300 cm), and 32 were males (body size range: 188–351 cm). Isolates showing phenotypical resistance to tetracycline were either directly obtained from supplemented media (20/53) or later confirmed after striking pure cultures on media supplemented with tetracycline (4/53), as was the case for the initial 16 samples. Tetracycline-resistant isolates were more frequently obtained from males (18/32) than females (6/21). Considering the management types, the detection rate of resistant *E. coli* differed between the animals liberated within the next 24 h after their capture in the ponds (10/33) and animals liberated from enclosures of the aquaculture production at the end of the year (14/20). According to the Fisher’s exact test, the latter difference was statistically significant (*p* = 0.0096). In contrast, neither sex nor age class seemed to statistically influence the detection frequency of tetracycline-resistant *E. coli* among the studied individuals (Fisher’s exact test *p* > 0.05). Specific data regarding the animals sampled and the isolates studied is summarized in Supplementary Table S1.

According to antimicrobial susceptibility testing, amikacin, cephalothin, cefazolin, cefepime, cefotaxime, ceftazidime, ceftriaxone, ciprofloxacin, colistin, fosfomycin, gentamicin, imipenem, ertapenem, meropenem, nitrofurantoin, and piperacillin/tazobactam effectively inhibited the growth of all *E. coli* isolates at the lowest concentrations tested. In contrast, resistance to tetracycline was confirmed in all isolates obtained from supplemented media, and further phenotypic resistance was observed in individual cases to ampicillin (5/24), ampicillin/sulbactam (2/24), nalidixic acid (1/24), and trimethoprim/sulfamethoxazole (1/24). Thus, mono-resistance to tetracycline was mainly observed among the resistant *E. coli* isolates (19/24). We did not observe microbial growth on media supplemented with cefotaxime (2 mg/L) from any samples. Phenotypic resistance profiles of tetracycline-resistant strains obtained from crocodiles captured in the premises of an aquaculture production are shown in Table 1.

**Table 1:** Phenotypic resistance profiles of tetracycline-resistant *E. coli* isolated from cloacal samples of American crocodiles (*C. acutus*) captured in an aquaculture production of the Northern Pacific region, Costa Rica.

Management type	Phenotypic resistance profile*	Number of isolates
Ponds	TET, AMP, SAM, NAL	1
	TET, AMP	3
	TET	6
Enclosure	TET, AMP, SAM, SXT	1
	TET	13

^*^AMP, ampicillin; SAM, ampicillin/sulbactam; NAL, nalidixic acid; TET, tetracycline; SXT, trimethoprim-sulfamethoxazole.

### 3.2 Whole-genome sequencing and phylogenetic analysis of tetracycline-resistant *Escherichia coli*

NextSeq 500 short-read, paired-end WGS results were subjected to the Assembly-based QUality Assessment for Microbial Isolate Sequencing pipeline (AQUAMIS)^1^ for quality evaluation. WGS generated 1.5 to 2.6 million reads per isolate gDNA sample, leading to *de novo* assemblies exhibiting a minimum sequencing depth of 45 per consensus base per genome. The individual *E. coli* genomes ranged between 4.56 and 5.10 Mbp, represented by contig numbers ranging between 127 and 232. The G + C content of the genomes ranges between 50.44 and 50.89%.

**Figure 1:**
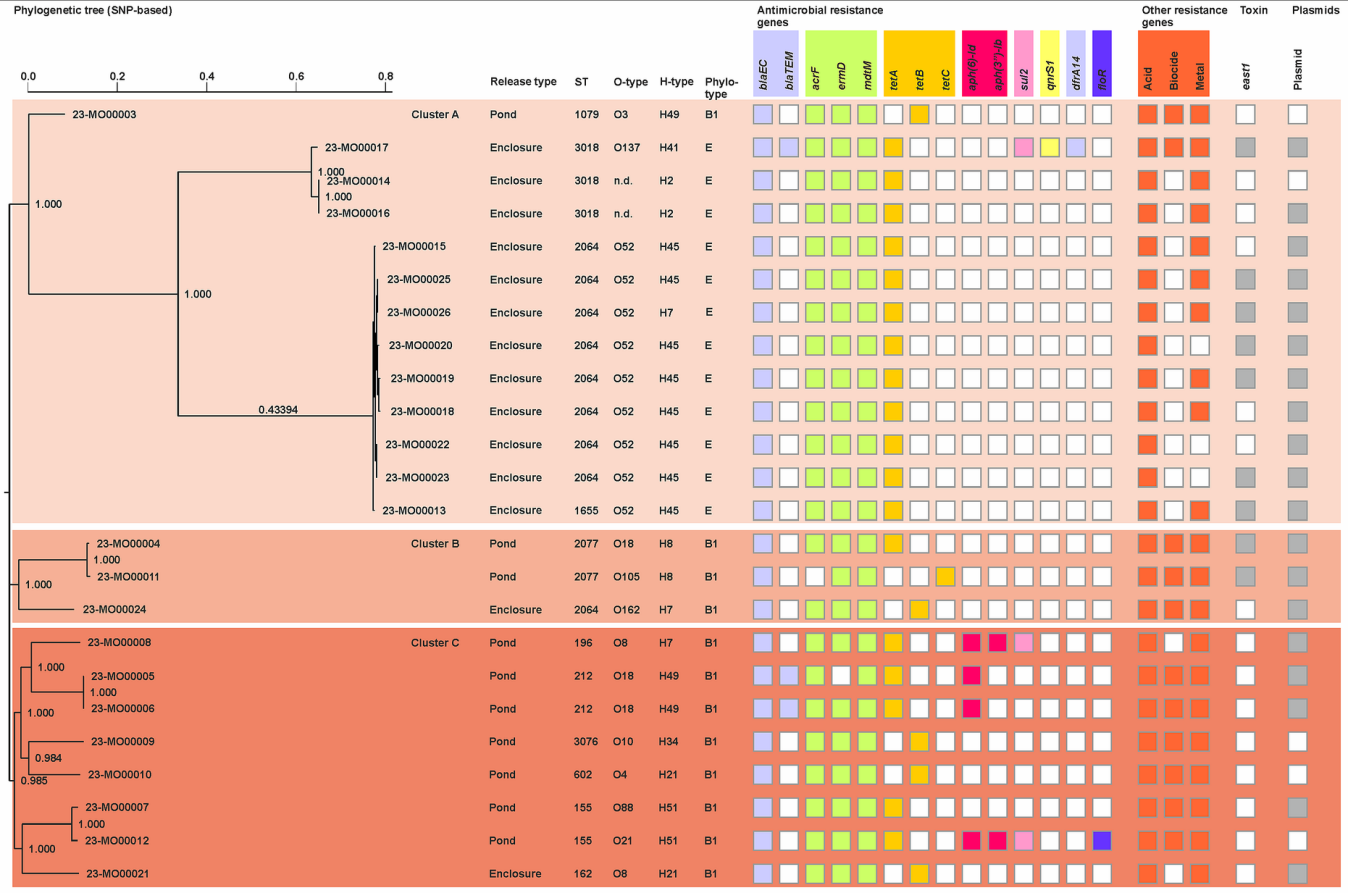
Hierarchical clustering analysis of tetracycline-resistant *E. coli* obtained from crocodiles captured in an aquaculture production of the Northern Pacific region, Costa Rica.

Phylogenetic relationships of the tetracycline-resistant *E. coli* from the crocodiles captured in the aquaculture production are shown in Figure 1. Analyses of the sequence data showed that tetracycline-resistant strains belonged only to the phylotypes B1 (12/24) or E (12/24). When considering the seven housekeeping genes (*adk, fumC, gyrB, icd, mdh, purA* and *recA*) used for multilocus sequence typing (MLST), resistant *E. coli*-isolates were assigned to 11 distinct sequence types (STs). Strains of the ST2064 were more frequently detected (9/24), followed by ST3018 (3/24), ST155 (2/24), ST196 (2/24), ST2077 (2/24), ST162 (1/24), ST602 (1/24), ST1079 (1/24), ST1665 (1/24), and ST3076 (1/24). Of note, all STs were either found in animals shortly released after capture or in those after longer enclosure (Figure 1). Additionally, separated by management type, tetracycline-resistant *E. coli* of animals kept for longer periods in the enclosure belonged to less different STs than those promptly released (14 isolates of 4 STs vs. 10 isolates of 7 STs). WGS analyses furthermore showed that *E. coli*-isolates belonged to serotypes OND:H2 (2/24), O6:H49 (1/24), O8:H7 (1/24), O8:H21 (1/24), O18:H49 (2/24), O52:H45 (9/24), O88:H51 (1/24), O105:H8 (1/24), O137:H41 (1/24), and O162:H7 (1/24), as well as the OND:H21 (1/24), OND:H34 (1/24), and 18:H8 (1/24). When considering both ST and serotype, it was possible to observe that the nine ST2064-isolates obtained from enclosed animals were assigned to O52:H45 (Figure 1). Notably, all isolates chromosomally harbored various genetic virulence factors and only three isolates from animals of the ponds additionally carried virulence genes on plasmids (Supplementary Table S1). Furthermore, gene *east1* encoding for the heat-stable toxin 1 was detected in 7 and 2 isolates obtained from males of the enclosure or females promptly removed from the aquaculture premises, respectively (Figure 1; Supplementary Table S1).

Exclusively considering the WGS data, three different clusters were generated based on SNP differences within the genome alignment (SNPA) (Figure 1). Out of the SNP tree, significant sequence similarities between the tetracycline-resistant strains obtained from animals held in captivity for more extended periods (Cluster A) were observed. The remaining strains were distributed in clusters A (1/24), B (3/24), and C (8/24).

### 3.3 Relationships between the phenotypic antimicrobial susceptibility profiles and carriage of resistance genes to acids, biocides, and metals

Phenotypic resistance to tetracycline was confirmed by the detection of either *tet*(A) (18/24), *tet*(B) (5/24), or *tet*(C) (1/24) genes. The two isolates, resistant to ampicillin/sulbactam and one of the ampicillin, carried *bla_TEM-1_*. Additionally, phenotypic resistance to nalidixic acid and trimethoprim/sulfamethoxazole was supported by detecting genes *gyrA_S83L* and *dfrA14*, respectively. Further resistance determinants were found for aminoglycosides (*aph(6)-Id*: 4/24; *aph(3″)-Ib*; 2/24), fosfomycin (*glpT_E448K*: 4/24), sulfonamides (*sul2*: 3/24), phenicol (*floR*: 1/24), quinolones (*qnrS1*: 1/24, *gyrA_S83L*: 1/24), and colistin (*pmrB_Y358N*: 1/24). With only a few exceptions, genes *acrF*, *emrD*, and *mdtM* encoding for efflux pumps were generally detected among the studied isolates (Figure 1).

Further gene sequences associated with resistance mechanisms to acids, biocides, and metals were detected among the tetracycline-resistant isolates. Generally, *E. coli* strains harbored genes *asr* (20/24), *ymgB* (24/24) encoding for resistance to acids, *emrE* (11/24) for biocide resistance, as well as *arsC* (20/24), *arsR* (14/24), and *fieF* (20/24) for metal resistance. In individual cases, *arsD*, *mer*-genes (*C, P*, *R*, and *T*), and *ter*-genes (*B, C*, *D,* and *E*) were also detected (Supplementary Table S1). Genes *pco* (*A, B, C, D, E, R,* and *S*), *sil* (*A*, *B*, *C*, *E*, *F*, *P*, *R*, and *S*) were together exclusively carried by the two ST212-strains of this study (Supplementary Table S1).

Regarding the plasmid carriage, 18 different sequences were determined in 19 *E. coli* isolates (Figure 1). Detected plasmids and localization of antimicrobial resistance genes are detailed in Supplementary Table S1. According to bioinformatic analyses, AMR genes of only seven isolates were plasmid-located, and only one of the latter strains was obtained from a male of the enclosure. Notably, genes *tet(A)* detected in the chromosome of 7 of the animals of the enclosure were located in unit transposon Tn1721. Furthermore, the two *E. coli* isolates ST212 obtained from animals of the ponds harbored all their resistance genes in a composite transposon carried by plasmid IncFIC(FII). Of note, plasmid ColE10 was exclusively detected in the ST2064 isolates of the animals temporarily held in captivity.

## 4 Discussion

Although genetic mechanisms responsible for AMR originate from environmental bacteria (46), an increase in the frequency of AMR detection and the complexity of resistance profiles has been widely reported in initially susceptible microbial populations, such as human and animal pathogens, in different natural ecosystems [e.g., review by (47)]. Our study analyzed the presence of AMR in a particular population of American crocodiles (*C. acutus*) in Costa Rica using *E. coli* as a microbiological indicator. Due to our sampling strategy, the last known location for the sampled individuals was the premises of a freshwater aquaculture production facility. Additionally, considering the management practices at this company, animals were differently exposed to the production environment, at least in terms of time. However, our sampling approach was convenient and limited to a one-year period. These aspects should be considered as limitations of the present study and thus, the degree of influence of factors related to the aquaculture farm on our observations cannot be measured with certainty. Furthermore, despite the use of cefotaxime for the isolation of resistant bacteria from the beginning of the study, not all samples were directly tested with tetracycline. Tetracycline was chosen because of its frequent use in aquaculture farms worldwide (48), the potential impact on the occurrence of AMR in the environment (49), as well as the application in Costa Rican agriculture of the Northwestern region (50). Tetracycline is authorized in Costa Rica for crops such as rice, banana, or beans (51). The delayed implementation of tetracycline supplementation may have affected the sensitivity of the overall study and an underestimation of the prevalence cannot be discarded. Considering these limitations, phenotypic antimicrobial resistance profiling and WGS do not outweigh the deficiencies but still expand the descriptive capacity of the present study. To the best of our knowledge, this is not only the first report describing AMR occurrence in crocodiles captured in the facilities of a freshwater-intensive aquaculture production but also the first report regarding the presence of AMR in *E. coli* obtained from free-living American crocodiles (*C. acutus*) in Mesoamerica.

Overall, microbiological analyses showed that the cloacal microbiota of *C. acutus* of the studied crocodiles contained tetracycline-resistant *E. coli.* Tetracycline-resistant *E. coli* were successfully isolated from animals. Detection was independent of sex or age of the sampled individuals. Additionally, through antimicrobial susceptibility testing, it was not only possible to confirm tetracycline resistance but also permitted the detection of further resistance to other antimicrobial compounds. Although tetracycline and its derivates are not currently a common target for the screening of AMR in wild populations, tetracycline resistance has been frequently reported in antimicrobial-resistant *E. coli* isolated from wild carnivorous species in America (15), Africa (6), and Europe (4, 5, 8, 11, 12, 14). By culture-independent methods, ARGs associated with tetracycline resistance were also detected in such wild populations (10, 17, 18). Current scientific evidence indicates that human activities are closely related to AMR occurrence in various ecological niches at different trophic levels (47). In this matter, studies of the last years support the hypothesis that the regional varying type, degree, and density of landscape’s anthropization differently shape AMR epidemiology in wild herbivore and omnivorous populations inhabiting the human-wildlife interface (52–57), which agrees with the observation in predators of such habitats (6, 10, 11, 17). Despite the relatively low settlement density, the habitats of the Costa Rican Northern Pacific region are characterized by the high density of crops and the presence of extensive livestock (27). Thus, it was expected to find, at least to some extent, antimicrobial-resistant *E. coli* in the sampled crocodiles. Interestingly, the management practices before release had a statistically significant effect on the detection frequency. Oxytetracycline is one of the main antibiotics used in Costa Rican aquaculture (50). By directly analyzing water and sediment samples, Seyfried et al. (49) observed that applying oxytetracycline in aquaculture increased the prevalence and diversity of resistance genes associated with tetracycline resistance in the surrounding water environment. Notably, ARGs for tetracycline were reported in an intensive aquaculture farm of the Northern Pacific region with a history of tetracycline application (58). Thus, the higher detection rate in animals in the enclosure is probably attributed to the longer stay in the aquaculture production environment. However, the occurrence of tetracycline resistance is not limited to aquaculture systems since its presence was previously documented in urban and agricultural runoffs (59, 60) as well as in livestock feedlots (61, 62). Such sources may be relevant in the studied region for the local crocodile specimens due to the solid agricultural character of the landscape (27) and the general use of tetracyclines in such production systems in Costa Rica (50).

Interestingly, phenotypic but particularly genotypic profiles also differed between both groups of the present study. Despite the higher detection frequency, it was observed that the *E. coli* isolates of the animals from the enclosure were more homogeneous than the strains of the animals liberated within 24 h after capture in the premises. This homogeneity was noticeable in the higher proportion of mono-resistant isolates, lower number of different STs and serotypes, and the grouping of most strains in cluster A (12/14). The most frequent *E. coli* strains were the mono-resistant ST2064 chromosomally carrying *tet*(A) with the serotype O52:H45 of phylogroup E, which was only detected among the crocodiles kept in captivity. Following the current scientific opinion, Brisson et al. (63) reported a higher probability of carrying antimicrobial-resistant *E. coli* for African herbivores kept in zoos than those of the same species sampled in natural parks. By non-cultural methods, apes in captivity showed more ARGs than their counterparts in wild environments (64). Such observations agree with previous observations in Costa Rica for the Central American tapir (Baird’s tapir; *Tapirus bairdii*). While a multidrug-resistant ESBL-producing *E. coli* was obtained from a captive individual (65), no resistant isolates were obtained in free-ranging Baird’s tapirs of Costa Rican protected mountainous areas (66).

In contrast with those reports, the comparison of ARGs occurrence between captive and wild Asian elephants (*Elephas maximus*) in China showed that the fecal microbiome of captive animals contained fewer ARG types than their wild relatives (67). Cao et al. (67) described that the wild elephants sampled during migration had a broader spectrum of exposure sources for ARGs than the captive animals of the same species. Based on the distribution and natural history of crocodiles in the Northern Pacific habitats (25–27), it is likely that crocodiles’ wandering through habitats with diverse types and degrees of anthropization favored their exposure to different sources of AMR before reaching the aquaculture premises. This behavior may explain the higher diversity of resistant *E. coli* strains observed after a short abode in the ponds. Thus, we hypothesized that the more prolonged captivity enables the colonization of crocodiles by the “resident” bacterial communities of the farm environment and results in the homogenization of *E. coli* strains and resistance patterns of their gastrointestinal microbiota with time. It is noteworthy, that the overall occurrence of tetracycline-resistant *E. coli,* along with the potential interactions with other several anthropogenic factors before reaching the facilities complicate definitive conclusions about the possible sources of antimicrobial resistance for the studied individuals. It is noteworthy, that these aspects have relevant implications for the possible role of American crocodiles as environmental “sentinels” in anthropized habitats.

Given the absence of regional epidemiological data on the prevalence of pathogens and resistant *E. coli*, it is challenging to elaborate further on the potential consequences of carriage of resistant *E. coli* by American crocodiles for the local communities, livestock or the environment. However, viewed through the lens of One Health, the presence of mono-and multiresistant *E. coli* carrying a variety of plasmid types and transposons within the examined crocodile individuals hold substantial significance. Due to their ability to move through different habitats, crocodiles establish extensive cross-ecosystem linkages, leading to several potential outcomes (22). For the environment, these reptiles’ carriage of farm-associated and other AMR and ARGs may have denigrative effects on the natural ecosystems. At least in theory, they may perpetuate the occurrence of antimicrobial-resistant bacteria and ARGs in natural habitats (47). This carriage would thus be especially relevant for individuals liberated in a National Park for nature conservation. Additionally, from a biosecurity standpoint, their role as carriers of potential pathogens (9, 31) and the “non-resident” AMR mechanisms may be relevant for health management in fish farms, as suggested for livestock (68). Considering the regional human-wildlife conflicts (e.g., bites) (28) and the potential transmission whether from or into aquaculture facilities, the detection of *E. coli* strains carrying genes for production of exotoxins, such as the enteroaggregative heat-stable toxin 1 (*east1*) (69), needs consideration by local communities and the national public health authorities.

## 5 Conclusion

This approach enabled the identification of tendencies related to the occurrence of tetracycline-resistant *E. coli* in American crocodiles from the Northern Pacific region in Costa Rica. Despite the limitations of the study, by combining cultural methods and WGS, we gained general insights into the potential implications of crocodile trespass in aquaculture environments, for both incoming individuals and the aquaculture production environment, in terms of carriage of tetracycline-resistant *E. coli*. Overall, the findings reveal that the occurrence of AMR and ARGs in American crocodiles is more complex and may not solely be attributed to the use of tetracycline substances in specific sectors, such as freshwater aquaculture farms. However, the widespread distribution of *C. acutus* and their ability to inhabit ecosystems with varying degrees of human impact may increase the risk and diversity of AMR carriage. Following the current knowledge about the spread of antimicrobial resistance in natural environments, disseminating such mechanisms in pathogens and commensal bacteria may pose diverse threats to public health, animal health, and ecosystems, at least at a regional level. Although human outbreaks caused by the detected *E. coli* strains have not, to the best of our knowledge, been reported in Costa Rica and the detected serotypes are not commonly enteropathogenic (70), our observations underscore the importance of implementing systematic surveillance of antibiotic resistance development in American crocodiles. The feasibility and scope of monitoring strategies for antimicrobial resistance in *C. acutus* and other crocodile species should be carefully evaluated by local authorities considering local characteristics such as landscape conditions, crocodiles’, human, and livestock densities, as well as probability of interactions (26–28).

## Data availability statement

The datasets presented in this study can be found in online repositories. The names of the repository/repositories and accession number(s) can be found in the article/Supplementary material.

## Ethics statement

The animal study was approved by local authorities within the framework of the management plan for crocodiles given in Resolución #M-P-SINAC-PNI-ACAT-041-2021. The study was conducted in accordance with the local legislation and institutional requirements.

## Author contributions

RM-V: Conceptualization, Data curation, Formal analysis, Visualization, Writing – original draft. VA-P: Formal analysis, Investigation, Validation, Writing – review & editing. IS-H: Conceptualization, Methodology, Resources, Supervision, Writing – review & editing. JH: Data curation, Formal analysis, Methodology, Resources, Visualization, Writing – review & editing. EB-C: Conceptualization, Data curation, Formal analysis, Methodology, Resources, Supervision, Writing – review & editing.

## Footnotes

^1^ https://gitlab.com/bfr_bioinformatics/AQUAMIS
